# Involvement of the Ventrolateral Prefrontal Cortex in Learning Others’ Bad Reputations and Indelible Distrust

**DOI:** 10.3389/fnhum.2016.00028

**Published:** 2016-02-04

**Authors:** Atsunobu Suzuki, Yuichi Ito, Sachiko Kiyama, Mitsunobu Kunimi, Hideki Ohira, Jun Kawaguchi, Hiroki C. Tanabe, Toshiharu Nakai

**Affiliations:** ^1^Department of Social and Human Environment, Graduate School of Environmental Studies, Nagoya UniversityNagoya, Japan; ^2^Neuroimaging & Informatics Laboratory, National Center for Geriatrics & GerontologyOhbu, Japan

**Keywords:** reputation, distrust, ventrolateral prefrontal cortex, cooperation, evaluation, learning, fMRI

## Abstract

A bad reputation can persistently affect judgments of an individual even when it turns out to be invalid and ought to be disregarded. Such indelible distrust may reflect that the negative evaluation elicited by a bad reputation transfers to a person. Consequently, the person him/herself may come to activate this negative evaluation irrespective of the accuracy of the reputation. If this theoretical model is correct, an evaluation-related brain region will be activated when witnessing a person whose bad reputation one has learned about, regardless of whether the reputation is deemed valid or not. Here, we tested this neural hypothesis with functional magnetic resonance imaging (fMRI). Participants memorized faces paired with either a good or a bad reputation. Next, they viewed the faces alone and inferred whether each person was likely to cooperate, first while retrieving the reputations, and then while trying to disregard them as false. A region of the left ventrolateral prefrontal cortex (vlPFC), which may be involved in negative evaluation, was activated by faces previously paired with bad reputations, irrespective of whether participants attempted to retrieve or disregard these reputations. Furthermore, participants showing greater activity of the left ventrolateral prefrontal region in response to the faces with bad reputations were more likely to infer that these individuals would not cooperate. Thus, once associated with a bad reputation, a person may elicit evaluation-related brain responses on their own, thereby evoking distrust independently of their reputation.

## Introduction

*Reputations*, information that signals the potential cooperativeness of others (Tennie et al., 2010), are known to facilitate cooperation (Milinski et al., 2002; Feinberg et al., 2014). Due to their impact, however, reputations are subject to distortion (e.g., spreading lies or gossip) by others seeking to flatter allies and tarnish rivals (Mayzlin, 2006). Although this fact underscores the importance of disregarding unfounded reputations, this ability appears limited. Bad reputations are difficult to ignore and can continue to affect one’s judgment even after they are shown to be false (Suzuki et al., 2013). The persistent effects of bad reputations are problematic given their power to cause avoidance (Chevalier and Mayzlin, 2006) and ostracism (Feinberg et al., 2014) of the target individuals. Elucidating the neural mechanisms underlying this persistence is therefore of theoretical and practical interest.

There has been growing interest in the neural basis of reputation processing (Frith and Frith, 2012; Izuma, 2012), and a few studies have investigated brain mechanisms underlying the difficulty of “unlearning” reputations through reinforcement learning based on social interactions. For example, Delgado et al. (2005) measured brain activity while participants were playing an iterated trust game and examined how it was modulated by the presence or absence of prior reputation about trading partners. When no reputation was available, the striatum showed greater responses to the partner’s cooperation than to cheating; when good or bad reputation was provided in advance, however, such differential striatal activity depending on the partner’s behavior diminished. These findings have been elaborated by Fouragnan et al. (2013) who conducted a similar experiment and analyzed the data using a computational model of reinforcement learning. They demonstrated that prior reputation about partners attenuated striatal activity in response to the trial-by-trial prediction error—the difference between the expected value of trusting a partner and the actual outcome from having trusted (i.e., cooperation [reward] or cheating [loss])—during a trust game.

In contrast, the neural underpinnings of the failure to intentionally disregard false reputations after being given verbal instructions that undermine their credibility (Suzuki et al., 2013) remain poorly studied. The present study approached this issue from the perspective of *evaluation transfer* in evaluative conditioning (Martin and Levey, 1978; Jones et al., 2009; Gawronski and Bodenhausen, 2011; but see also Hofmann et al., 2010). This refers to the transfer of the evaluation from an unconditioned stimulus (US) to a conditioned stimulus (CS).^1^

For instance, suppose you are told that Ken, a bank employee, embezzled money from client accounts. A subsequent encounter with Ken will remind you of his embezzlement, and you are likely to conclude that Ken is untrustworthy because of his cheating behavior. In addition, reputation learning may also cause the transfer of the evaluation from the reputation (US) to the target individual (CS) such that the person acquires an ability to activate a positive or negative evaluation on their own. That is, the negative evaluation made about embezzlement becomes associated with Ken himself and consequently Ken alone generates a negative evaluation.

In neural terms, evaluation transfer would be operationalized as the CS alone activating evaluation-related brain structures. In general, item evaluation is considered to be an essential component of emotional processing (Russell, 2003). Thus, many cortical and subcortical structures linked with emotions are assumed to be involved in the evaluation of stimuli, including ventral portions of the prefrontal cortex (Kringelbach and Rolls, 2004), anterior parts of the insular (Singer et al., 2009) and cingulate cortices (Rushworth and Behrens, 2008), the amygdala (Morrison and Salzman, 2010), and the striatum (O’Doherty, 2004). Of particular relevance to reputation learning, these neural structures have been implicated in the evaluation of others’ behavior in previous studies (Sanfey et al., 2003; Delgado et al., 2005; Buckholtz et al., 2008; Rilling et al., 2008; Schiller et al., 2009; Mende-Siedlecki et al., 2013). Thus, after learning one’s reputation, the target individual may activate the evaluation-related brain region on his/her own, constituting a neural correlate of evaluation transfer. More specifically, with regard to bad reputations, the involvement of the lateral and ventral portions of the prefrontal cortex and the anterior insula might be expected since these regions have been implicated in anger and disgust (Murphy et al., 2003; Vytal and Hamann, 2010; but see also Lindquist and Barrett, 2012), which are negative emotions closely related to appraisals of harmfulness and immorality (Hutcherson and Gross, 2011).

A hallmark of evaluation transfer is the difficulty with which one can intentionally negate its effects (Gawronski and Bodenhausen, 2011). Suppose that Ken’s reputation as an embezzler is later found to be invalid. Then, you will not reason that Ken is untrustworthy from the inaccurate reputation that he embezzled money. However, if evaluation transfer has occurred, Ken will still elicit negative evaluations independently of the validity of his reputation, and therefore, will continue to be distrusted. It has indeed been reported that the effect of evaluation transfer cannot be neutralized voluntarily (Sweldens et al., 2010; Balas and Gawronski, 2012), and that negative evaluations are especially transferable (Rydell and Jones, 2009; Bell et al., 2012; Campbell and Warren, 2012). It can therefore be hypothesized that the persisting effects of a bad reputation are related to a transfer of the negative evaluation about the bad reputation to the target person. Here, we report a functional magnetic resonance imaging (fMRI) experiment testing this hypothesis.

In this fMRI study, participants memorized faces paired with either a good or a bad reputation. Next, they viewed the faces alone and inferred whether each person would be likely to cooperate, first while retrieving the memorized reputations and then while trying to disregard them as false. If reputation learning transfers the negative evaluation of bad reputations to target persons, and if the transfer is related to the persisting distrust, the following two predictions would be made. **Prediction 1**: Face stimuli that are paired with bad reputations during a learning task will activate an evaluation-related brain region during an inference task, irrespective of whether participants attempt to retrieve or disregard the reputations. **Prediction 2**: Participants showing higher activity of the region described in Prediction 1 will infer that the persons with bad reputations are less likely to cooperate, irrespective of whether participants attempt to retrieve or disregard the reputations.

## Materials and Methods

### Participants

Thirty-two undergraduate and graduate students (18 males and 14 females; age 20–31 years) gave informed consent to participate in this study, which was approved by the Ethics Committees of the National Center for Geriatrics and Gerontology, Japan, and the Graduate School of Environmental Studies, Nagoya University, Japan. Four participants (2 males and 2 females) were excluded from analyses because one withdrew due to fatigue, two expressed suspicion about a cover story for the experiment, and one showed perfect performance in the good-reputation condition of the baseline session of action inference.^2^^,^^3^

### Stimuli

Twenty-four neutral faces of Japanese individuals (12 males and 12 females) from the Facial Information Norm Database (Watanabe et al., 2007) were used as stimuli. They were divided into three groups and each was assigned to one of the three conditions: *good-*, *bad-*, and *no-reputation*. The assignment of reputations to faces was counterbalanced across participants. The three groups of faces were matched for number of males and females and mean trustworthiness rating (1, *very untrustworthy*, to 5, *very trustworthy*) assessed in a preliminary survey with 102 participants (*M* = 2.96, 2.96, 2.97; unpublished data). All face stimuli were presented in gray scale and were cropped into square shapes (270 × 270 pixels) so that only the central facial features (eyes, eyebrows, nose, and mouth) were visible.

### Experimental Procedure

**Figure 1:**
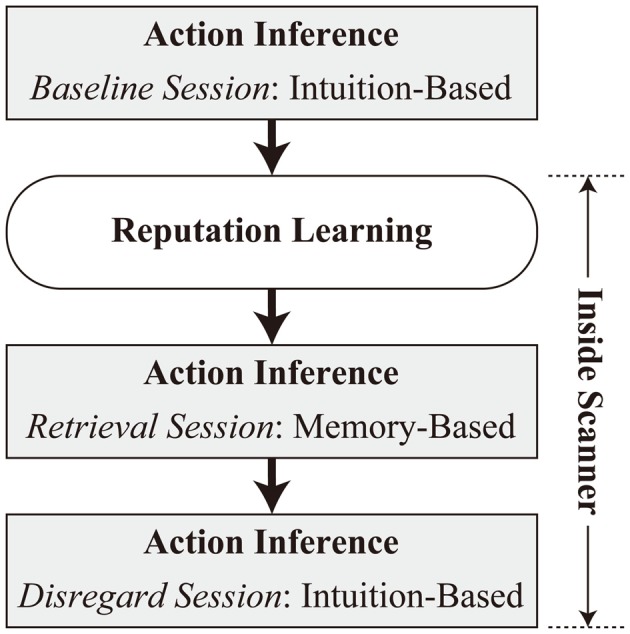
**Overview of the experimental procedure**.

The experiment consisted of four tasks in the following order: baseline session of action inference, reputation learning, retrieval, and disregard sessions of action inference (Figure 1). The baseline session of action inference was performed on a laptop computer (HP ProBook 4740s, Hewlett-Packard Japan, Ltd., Tokyo, Japan) outside an MRI scanner. The other tasks were administered inside the scanner, with a short rest outside of the scanner after completion of the reputation-learning task.^4^ Inside the scanner, stimuli were presented with VisuaStim digital goggles (Resonance Technology, Inc., Northridge, CA, USA), and responses were collected via bimanual response pads (Current Designs, Inc., Philadelphia, PA, USA). Throughout the experiment, E-Prime 2.0 (Psychology Software Tools, Inc., Pittsburgh, PA, USA) was used to run the task. The display resolution was set to 800 × 600 pixels.

#### Baseline Session of Action Inference

Participants were presented with a cover story about the experiment. They were instructed that they would see the faces of unfamiliar people who had taken part in a two-player “investment game” in a previous (fictitious) study. It was explained that in this game, one player (“lender”) is provided with 500 yen (about 5 USD) and decides whether to invest the money in the other player (“borrower”). When 500 yen is invested, the money is quadrupled to 2000 yen, and the borrower decides whether to return a 1000 yen dividend to the lender or to embezzle the whole amount. Participants in the current study were informed that the persons whose faces they would see during the present experiment had played the investment game as a borrower previously, and had received a 500 yen investment from their partners. The participants’ task was to infer intuitively, based on their impressions of the faces, whether each person had returned the dividend (pressing the “F” key with their left index finger) or embezzled the investment (pressing the “J” key with their right index finger). The faces were presented until participants responded with no time limit. The task was repeated in two blocks, and each of the 24 faces was presented once per block.

#### Reputation Learning

**Figure 2:**
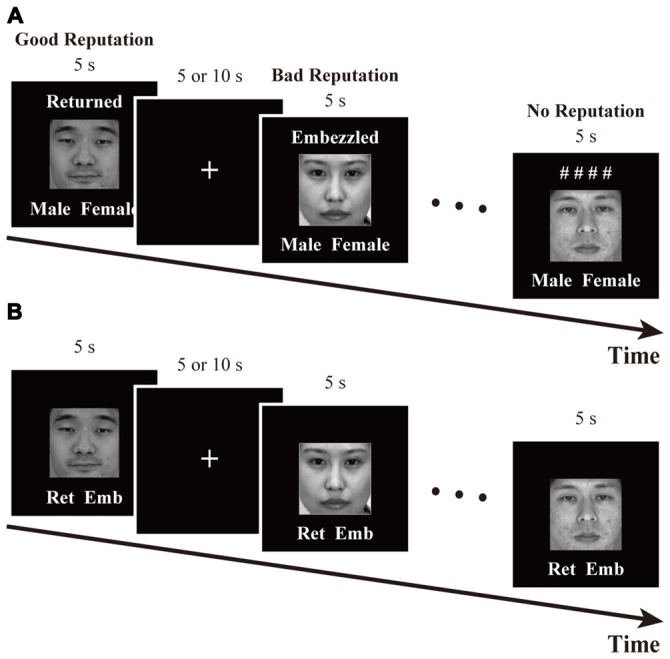
**Time courses of (A) the reputation-learning task and (B) the action-inference task**. Labels were written in Japanese during the experiments: Returned = 
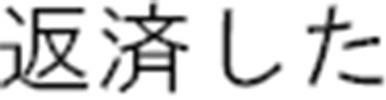
; Embezzled = 
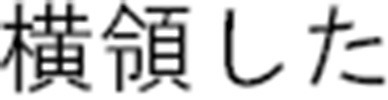
; Male = 
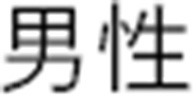
; Female = 
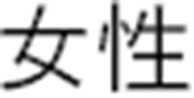
; Ret = 
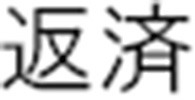
; Emb = 
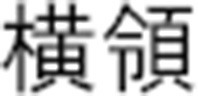
.

The time course of this task is schematized in Figure 2A. In each trial, a face was presented for 5 s along with one of the following labels: “Returned,” “Embezzled,” and “# # # #.” The words “Returned” and “Embezzled” ostensibly indicated that the person had returned (*good-reputation condition*) and embezzled (*bad-reputation condition*) the investment in the previous experiment. The symbolic label of “# # # #” indicated that whether the person had returned or embezzled was being kept confidential (*no-reputation condition*). Below each face were also displayed the words “Male” and “Female.” Participants were instructed to memorize whether the displayed person had returned or embezzled the money, while concurrently indicating whether they were male (pressing a left-hand pad with their index finger) or female (pressing a right-hand pad with their index finger) as soon as possible. Sex identification was imposed in order to maintain participant attention.

The task was comprised of three fMRI runs that lasted for 305 s each. Each run started and ended with fixation periods that lasted for 20 and 15 s, respectively, and included 24 trials in between. Each of the 24 faces appeared once per run, and their presentation order was randomized. Between any two trials was a fixation interval the duration of which was randomly set to either 5 or 10 s, with a mean of 6.25 s.

#### Retrieval Session of Action Inference

The time course of this task is schematized in Figure 2B. In each trial, a face was presented for 5 s along with the abbreviated labels for “Returned” and “Embezzled” at the bottom. The participants’ task was to answer as quickly and as accurately as possible whether the person had returned (pressing a left-hand pad with their index finger) or embezzled (pressing a right-hand pad with their index finger) the money by recalling the reputations memorized during the previous task. For individuals with no reputation, participants were asked to make intuitive, face-based judgments, just as during the baseline session.

The task was comprised of two fMRI runs that lasted for 305 s each. Each of the 24 faces appeared once per run, and their presentation order was randomized. The durations of the fixation periods at the start and end of each run and between trials were the same as those in the reputation-learning task.

#### Disregard Session of Action Inference

The time course of this task was the same as that of the retrieval session of action inference. Prior to the task, an apology was given to participants, explaining that although the stimulus persons had previously taken part in the investment game as a borrower, the previously presented reputations about them were completely unrelated to their actions. Then, participants were told to disregard these invalid reputations and perform the action inference task again on the basis of their impressions of the faces. The task was comprised of two fMRI runs, and the timeline of each run was the same as that of the retrieval session.

After the completion of this task, participants were asked if they had any doubts regarding the experimental procedure. Two participants spontaneously mentioned their suspicions about the cover story that the stimulus individuals had previously taken part in the investment game, and thus their data were excluded from analyses.

### Imaging Protocol

MR images were acquired on a 3T scanner (Siemens MAGNETOM Trio, Erlangen, Germany) with a 12-channel head coil. Functional images were acquired using a T2*-weighted gradient echo planar imaging sequence with the following parameters: repetition time (TR) = 2500 ms, echo time (TE) = 30 ms, flip angle (FA) = 90°, matrix 64 × 64, field of view (FOV) = 192 mm, 39 slices, slice thickness = 3 mm, distance factor = 17%, and slice acquisition order = ascending. Following the completion of the disregard session of action inference, a high-resolution, magnetization-prepared, rapid-acquisition gradient echo (MPRAGE) image was also acquired for anatomical details (TR = 2500 ms, TE = 2.63 ms, FA = 7°, matrix 256 × 256, FOV = 256 mm, 208 slices per slab, slice thickness = 1 mm, and distance factor = 50%).

### Image Preprocessing

Reputation learning, retrieval, and disregard sessions of action inference involved three, two, and two functional runs, respectively. In each run, 122 functional images were acquired, of which the first two images were discarded to allow for T1 equilibrium. The remaining functional images were preprocessed with Statistical Parametric Mapping 8 (SPM8, Wellcome Trust Centre for Neuroimaging, London, UK) implemented in MATLAB R2013a (The Mathworks, Inc., Natick, MA, USA). For each participant, the images from all tasks and runs were realigned and resliced to the mean image to correct for head movement. Slice-timing correction was also performed using the twentieth slice as a reference. All functional images and the MPRAGE anatomical image were then co-registered to the mean image of the retrieval session of action inference. The co-registered anatomical image was normalized to a standard T1 template image (ICBM 152), which defined the Montréal Neurological Institute (MNI) space. The parameters from this normalization process were then applied to all functional images. Finally, the normalized functional images were spatially smoothed with an isotopic Gaussian kernel of 6 mm full-width at half-maximum.

### fMRI Data Analysis

Data from the fMRI were analyzed using SPM8. To depict the neural substrates of the tasks, we employed a summary statistics approach. In the individual-level analysis, a voxel-by-voxel general linear model (GLM) was applied to preprocessed functional images for each of the three in-scanner tasks separately. The design matrix of the model contained three regressors of interest (good-, bad-, and no-reputation conditions) to obtain parameter estimates for each reputation condition. The regressors of interest were created by convolving a delta function (0 s duration), representing trial onset times of each reputation condition, with a canonical hemodynamic response function (Friston et al., 1994). Moreover, when analyzing the reputation-learning data, parametric modulation regressors were included in the good- and bad-reputation conditions to remove the effect of the unexpectedness of the reputation. For example, for each reputation-learning trial of the good-reputation condition, the unexpectedness of the good reputation was computed as follows: Unexpectedness = Number of “Embezzled” responses for the displayed person in the baseline session of action inference (i.e., 0, 1, or 2) × 0.5^Run number − 1^. That is, we assumed a decrease of the unexpectedness across runs.^5^ The vector of the computed values was entered as parametric modulation regressors for the good-reputation condition. The unexpectedness of the bad reputation was computed in the same way by using the number of “Returned” responses.

In the group-level analyses, we first explored brain regions that were activated in response to good and bad reputations during the reputation-learning task. The individual-level analysis of the reputation-learning data produced the contrast images from each of the good- and bad-reputation conditions, where the effect of the unexpectedness of the reputation was controlled for, as well as the contrast images from the no-reputation condition. These individual-level contrast images for each reputation condition during reputation learning were submitted to random-effects GLM analysis. The design matrix contained three regressors of interest (good-, bad-, and no-reputation conditions) to obtain parameter estimates for each reputation condition, as well as each participant’s mean response times (RTs) in each condition as covariates to accommodate RT differences between conditions (see “Behavioral Data” Section).

Then, clusters of voxels that were significantly active in the contrast images of good vs. no reputation and bad vs. no reputation were identified as the regions of interest (ROIs) likely related to positive and negative evaluation, respectively. The statistical threshold was set at *p* < 0.05 (family-wise error [FWE] corrected) at the voxel level with no less than 20 contiguous voxels.

In order to test Prediction 1, we examined whether the negative-evaluation-related ROIs were significantly activated by the faces with bad reputations during the retrieval and disregard inference sessions.^6^ For this, ROI analysis was performed on the data from each session using MarsBaR toolbox for SPM (Brett et al., 2002). Specifically, parameter estimates were extracted and averaged across voxels in each ROI. The design matrix was the same as the one used in the analysis of the reputation-learning data. We tested whether each ROI activity, defined as the mean of the voxel values within it, was significantly greater in the bad-reputation cases as compared to the no-reputation cases. Similar analyses were also conducted for positive-evaluation-related ROIs.

In order to test Prediction 2, we examined the relationship between the activity of each negative-evaluation-related ROI and behavioral inferences during the retrieval and disregard sessions. Linear mixed-model analysis (West et al., 2007) was performed to achieve this goal. We examined whether post-learning distrust toward the people with bad reputations (DISTRUST_POST) was statistically explained by pre-learning distrust (DISTRUST_PRE) and the activation of the ROI toward those people (ROI_ACT). To allow for different effects of pre-learning distrust and ROI activity across sessions, we fit two models, one without and the other with interaction terms involving the session, and then selected the best-fit model. Specifically, the following two nested models were compared:

Model 1 (without interaction terms):

$$\begin{array}{l} \text{DISTRUST}\_{\text{POST}}_{si}\\ \;=\gamma_{00}+\gamma_{0\text{1}}\times {\text{SESS}}_{s}+\gamma_{\text{1}0}\times \text{DISTRUST}\_{\text{PRE}}_{i}\\ \;+\gamma_{\text{2}0}\times \text{ROI}\_{\text{ACT}}_{si}+u_{i}+\varepsilon_{si}. \end{array}$$

Model 2 (with interaction terms):

$$\begin{array}{l} \text{DISTRUST}\_{\text{POST}}_{si}\\ \;=\gamma_{00}+\gamma_{0\text{1}}\times {\text{SESS}}_{s}+\gamma_{\text{1}0}\times \text{DISTRUST}\_{\text{PRE}}_{i}\\ \;+\gamma_{\text{2}0}\times \text{ROI}\_{\text{ACT}}_{si}\\ \;+\gamma_{\text{11}}\times {\text{SESS}}_{s}\times \text{DISTRUST}\_{\text{PRE}}_{i}\\ \;+\gamma_{\text{21}}\times {\text{SESS}}_{s}\times \text{ROI}\_{\text{ACT}}_{si}+u_{i}+\varepsilon_{si}. \end{array}$$

The subscript *s* refers to the session of action inference (*s* = 1: retrieval session, *s* = 2: disregard session), and the subscript *i* denotes the *i*-th participant (*i* = 1, …, 28). The dependent variable DISTRUST_POST_*si*_ indicates each participant’s post-learning distrust toward the people that had been paired with a bad reputation, calculated as the difference in rate of judging that people had returned the investment between the no-reputation and bad-reputation conditions. A positive value of DISTRUST_POST_*si*_ means that the people with bad reputations were judged as *unlikely* to have returned the money. The same difference in the baseline session of action inference, labeled as DISTRUST_PRE_*i*_, was entered as a regressor to account for pre-learning distrust toward the people with bad reputation. ROI_ACT_*si*_ was the main regressor, representing each participant’s activation of the ROI in response to the people with bad reputations (i.e., contrast estimate of bad- vs. no-reputation conditions) in each session. The other regressor, SESS_*s*_, was a dummy variable indicating the session of action inference, with 0 and 1 for the retrieval and disregard sessions, respectively (i.e., SESS_1_ = 0 and SESS_2_ = 1). *γ*’s were fixed-effect parameters to be estimated. *u_i_* and *ε_si_* indicate random effects associated with each participant and each observation, respectively. They were assumed to be independently and normally distributed with a mean of 0 and variance of $\sigma_{u}^{2}$ or *σ^2^*. The random effects of *u_i_* were included to account for the dependency between sessions due to repeated measurements of the same participants (Aarts et al., 2014).

With regard to the differences between the two models, Model 1 assumes that while the intercept of the regression model may vary between sessions (i.e., *γ*_00_ + *γ*_01_ × SESS_*s*_),^7^ the fixed effects of pre-learning distrust (*γ*_10_) and ROI activity (*γ*_20_) on post-learning distrust are common across sessions. On the other hand, Model 2 allows for different fixed effects of pre-learning distrust and ROI activity across sessions (*γ*_11_ and *γ*_21_, respectively) by including interaction terms, SESS × DISTRUST_PRE and SESS × ROI_ACT.

The fits of the two models to the data were compared using Akaike and Bayesian information criteria (AIC and BIC, respectively) as well as a deviance test (West et al., 2007; Snijders and Bosker, 2012). AIC and BIC are measures of the fit of data to a model (smaller values indicate a better fit) with a penalty for increased model complexity. Deviance is also a goodness-of-fit index but without a penalty for complexity; therefore, its value is *always* smaller (indicating a better fit) for a more complex model. The difference between the deviance scores of two nested models can be statistically tested because it is distributed asymptotically as chi-squared under the null hypothesis of no difference. A significant difference supports the more complex model, while nonsignificance favors the simpler, more parsimonious model. Thus the information criteria and deviance tests compensate for each other’s weaknesses (i.e., lack of a statistical test and insensitivity to model complexity).

Model estimation was conducted using the lme4 (Bates et al., 2014) and lmerTest (Kuznetsova et al., 2014) R packages (R version 3.0.2). All continuous variables (i.e., DISTRUST_POST, DISTRUST_PRE, ROI_ACT) were centered and scaled before analysis so that standardized fixed-effect parameters were obtained.

## Results

### Behavioral Data

#### Sex Identification During Reputation Learning

The accuracy of sex identification during the reputation learning task (*M* ± *SD* = 0.894 ± 0.048) did not significantly differ between the reputation trials, *F*_(2,54)_ = 0.003, *MSE* = 0.013, *p* = 0.997, $\eta_{p}^{2}$ < 0.001. The RT of sex identification significantly varied based on reputation, *F*_(2,54)_ = 9.573, *MSE* = 0.115, *p* = 0.003, $\eta_{p}^{2}$ = 0.262, and was shorter in the no-reputation trials, *M* ± *SD* = 1.245 ± 0.346 s, than in both the good-, *M* ± *SD* = 1.518 ± 0.621 s, *t*_(27)_ = 3.213, *p* = 0.003, and bad-reputation trials, *M* ± *SD* = 1.495 ± 0.616 s, *t*_(27)_ = 3.136, *p* = 0.004.

#### Action Inference

**Figure 3:**
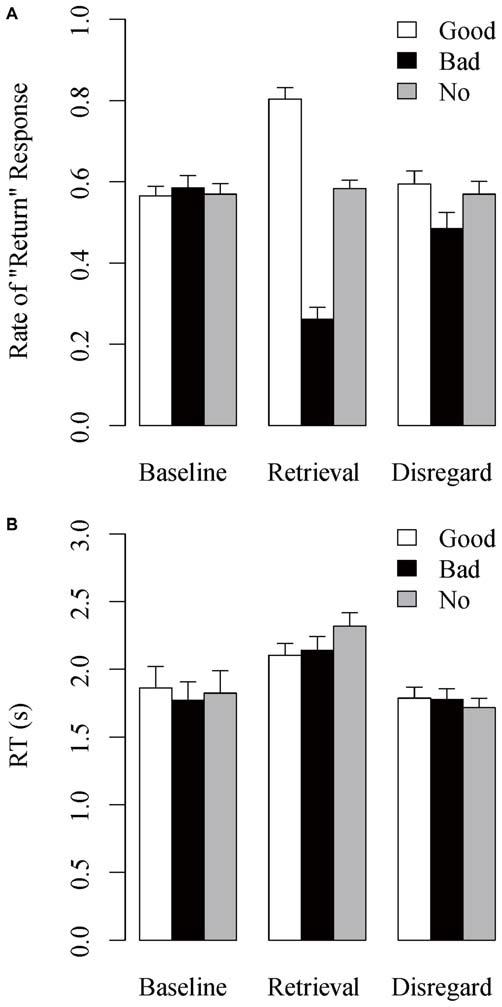
**Mean (A) rate of “return” response and (B) RT in the action-inference tasks as a function of reputation condition and task session**. Error bars indicate standard errors of the means.

Figure 3A shows the mean rate of “return” response, the rate at which the stimulus individuals were judged as having returned the investment, as a function of reputation condition and task session. The Reputation × Session interaction was significant, *F*_(4,108)_ = 42.470, *MSE* = 0.016, *p* < 0.001, est $\eta_{p}^{2}$ = 0.611. *Post hoc* analyses showed that the main effect of Reputation was not significant in the baseline session, *F*_(2,54)_ = 0.248, *MSE* = 0.013, *p* = 0.777, est $\eta_{p}^{2}$ = 0.009, whereas it was significant in the retrieval session, *F*_(2,54)_ = 94.539, *MSE* = 0.026, *p* < 0.001, est $\eta_{p}^{2}$ = 0.778, and in the disregard session, *F*_(2,54)_ = 3.855, *MSE* = 0.024, *p* = 0.027, est $\eta_{p}^{2}$ = 0.125. In the retrieval session, the positive difference of rate of “return” response in the good-reputation minus no-reputation trials was significant, *t*_(27)_ = 6.211, *p* < 0.001, as well as the negative difference in the bad- minus no-reputation trials, *t*_(27)_ = 9.221, *p* < 0.001, indicating the overall success of reputation learning. In the disregard session, the negative difference in the bad- minus no-reputation trials remained marginally significant, *t*_(27)_ = 2.013, *p* = 0.054, whereas the positive difference in the good-reputation minus no-reputation trials did not, *t*_(27)_ = 0.606, *p* = 0.549, replicating the persisting effect of bad reputations (Suzuki et al., 2013).

Figure 3B shows the mean RT in the action-inference task as a function of reputation condition and task session. A Reputation × Session interaction was significant, *F*_(4,108)_ = 5.380, *MSE* = 0.047, *p* = 0.002, est $\eta_{p}^{2}$ = 0.166. *Post hoc* analyses showed that the main effect of Reputation was not significant in either the baseline, *F*_(2,54)_ = 1.708, *MSE* = 0.037, *p* = 0.192, est $\eta_{p}^{2}$ = 0.060, or disregard sessions, *F*_(2,54)_ = 1.560, *MSE* = 0.027, *p* = 0.220, est $\eta_{p}^{2}$ = 0.055, whereas it was significant in the retrieval session, *F*_(2,54)_ = 8.075, *MSE* = 0.047, *p* = 0.001, est $\eta_{p}^{2}$ = 0.230. In the retrieval session, RT was significantly longer in the no-reputation trials than in both the good-reputation, *t*_(27)_ = 3.422, *p* = 0.002, and the bad-reputation trials, *t*_(27)_ = 3.248, *p* = 0.003. In addition, across reputation conditions the retrieval-session RT was longer than the baseline- and disregard-session RTs (all *p*’s < 0.10).

### fMRI Data

#### Reputation Learning

**Figure 4:**
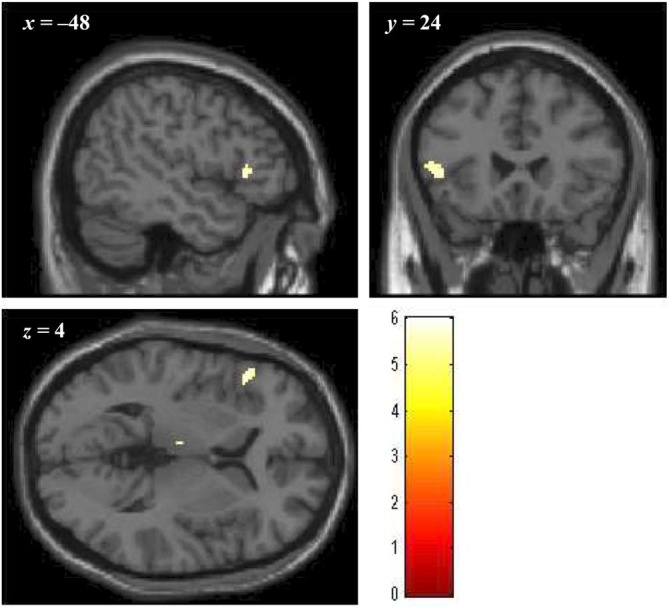
**The cluster of voxels in the vlPFC region significantly activated in response to bad reputations during reputation learning (*p* < 0.05, FWE corrected at the voxel level; minimum cluster size 20 voxels)**. Numbers above slices indicate coordinates in Montréal Neurological Institute (MNI) space.

Brain regions showing significantly greater activation in the bad- as compared to the no-reputation conditions during reputation learning (*p* < 0.05, FWE corrected at the voxel level; minimum cluster size 20 voxels) were the left ventrolateral prefrontal cortex (vlPFC; cluster size = 47 voxels, peak coordinates = [−48, 24, 4], *Z* = 5.258) and the left thalamus (cluster size = 31 voxels, peak coordinates = [−4, −16, 8], *Z* = 5.445). As described in the “Introduction” Section, ventral portions of the prefrontal cortex are implicated in the evaluation of stimuli, and, especially, the lateral regions are responsive to negative stimuli (Murphy et al., 2003; Kringelbach and Rolls, 2004; Elliott et al., 2010; Vytal and Hamann, 2010). Thus, the vlPFC activity likely reflects the negative evaluation of bad reputations. In contrast, the activity in the thalamus might reflect enhancement of perceptual processing by negative stimuli (Vuilleumier, 2005). We thus defined the left vlPFC region as the negative-evaluation-related ROI (Figure 4). With regard to the good-reputation trials, no brain region showed significantly greater activation as compared to the no-reputation trials. In addition, no brain region showed significantly greater activation for bad than for good reputations or* vice versa*.

**Figure 5:**
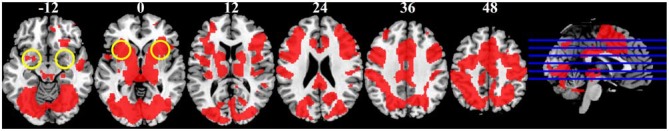
**Regions significantly active across the three reputation conditions during reputation learning identified by conjunction analysis (*p* < 0.001, uncorrected for multiple comparisons; shown in red)**. Numbers above slices indicate *z* coordinates in MNI space. Yellow circles at *z* = −12 and *z* = 0 indicate approximate locations of the amygdala and the anterior insula, respectively.

Contrary to our expectation, the anterior insula did not show greater activity in response to bad as compared to no reputation. Instead, this region was activated across reputation trials (Figure 5). The anterior insular activity in the no-reputation trials might make sense considering that participants were not told whether the stimulus individuals had returned or embezzled the money in this condition. Thus, although speculative, participants might have perceived these trials as risky (e.g., the odds of having returned or embezzled were 50/50) or ambiguous (i.e., the odds were completely unknown), thereby activating the anterior insula (Singer et al., 2009).

We also explored brain regions whose activity increased with the degree of unexpectedness of the reputation. Although with a less stringent threshold compared to the analysis above (*p* = 0.001 at the voxel level, uncorrected; minimum cluster size = 20 voxels), such a trend was detected in the bilateral middle cingulate cortices (left: cluster size = 20 voxels, peak coordinates = [−10, 8, 46], *Z* = 3.920; right: 21 voxels, [14, 4, 48], *Z* = 4.154) and right middle temporal region (71 voxels, [56, −34, −2], *Z* = 3.963), which were shown to be sensitive to the need for performance adjustment (Ridderinkhof et al., 2004) and the error in predicting others’ behaviors (Behrens et al., 2009), respectively. The bilateral fusiform gyri (left: 21 voxels, [−28, −54, −14], *Z* = 3.526; right: 33 voxels, [30, −36, −10], *Z* = 3.574) showed the same trend as well.

#### Action Inference

The mean activity of the voxels in the vlPFC ROI (Figure 4) was greater for the faces that had been paired with a bad reputation than it was for those with no reputation. This was marginally significant in the retrieval session (contrast value = 0.414, *Z* = 1.736, *p* = 0.087) and significant in the disregard session of action inference (contrast value = 0.707, *Z* = 2.717, *p* = 0.007). The mean vlPFC activity in the bad- vs. no-reputation conditions did not differ significantly between the retrieval and disregard sessions (*p* = 0.338), and its average across the two sessions was significantly greater than zero (*p* = 0.006).^8^ In addition to the ROI analysis, we conducted whole-brain conjunction analysis to explore regions showing greater activation in the bad- than no-reputation conditions both during reputation learning and during the two sessions of action inference. With a lenient statistical threshold (*p* = 0.05 at the voxel level, uncorrected; minimum cluster size = 20 voxels), this analysis also identified the vlPFC (cluster size = 100 voxels, peak coordinates = [−48, 24, 4], *Z* = 2.390).^9^ Overall, these results support Prediction 1. The activity in the left thalamus showing significantly greater responses in the bad- vs. no-reputation conditions during reputation learning was also subjected to the same ROI analysis, yielding non-significant results.

**Figure 6:**
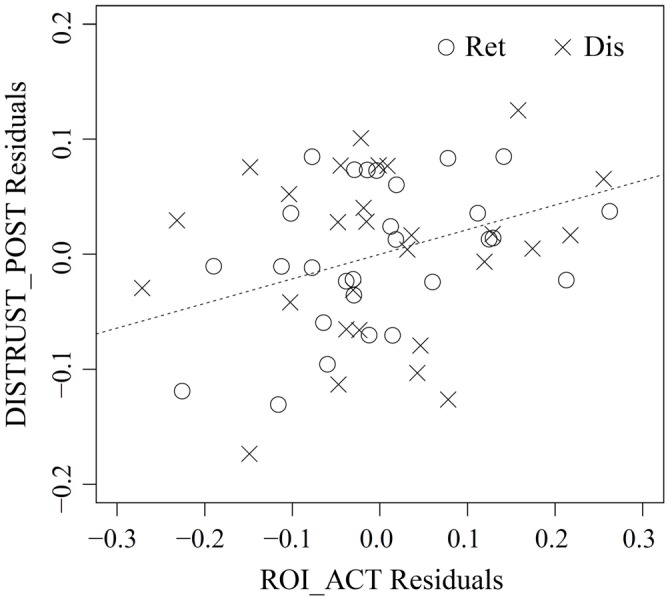
**Partial regression plot between vlPFC activity in response to the people with bad reputation (ROI_ACT) and distrust toward them (DISTRUST_POST) in the retrieval (Ret) and disregard (Dis) sessions of action inference**. The abscissa represents the residuals from the regression of ROI_ACT on DISTRUST_PRE and SESS, whereas the ordinate represents the residuals from the regression of DISTRUST_POST on the same two variables. The plot thus illustrates the marginal relationship between ROI_ACT and DISTRUST_POST after the effect of the other variables has been removed (Faraway, 2005). For reference, the dashed line shows DISTRUST_POST Residuals = 0.214 (point estimate of *γ*_20_ from Table 2) × ROI_ACT Residuals. See “fMRI Data Analysis” Section for more detailed descriptions of each variable.

As no reputation was displayed during action inference, the abovementioned vlPFC activity in the bad-reputation condition suggests that the stimulus faces themselves might have acquired the capacity to elicit negative evaluation directly. In order to verify this possibility, linear mixed-model analysis was performed to examine whether vlPFC activity in response to the faces paired with bad reputations could explain distrust toward them (i.e., the lower rate of “return” responses). Table 1 compares the goodness of fit of the two regression models, Model 1 and Model 2, without and with an interaction term allowing for different relationships between vlPFC activity and distrust in the retrieval and disregard sessions (see “fMRI Data Analysis” Section for details of the models). Model 1 was selected because of its superiority in terms of both information criteria and model parsimony (West et al., 2007). Table 2 summarizes the parameter estimates for Model 1, demonstrating that vlPFC activity was a significant predictor of distrust. That is, during both the retrieval and disregard sessions of action inference, participants who showed larger vlPFC activity in response to faces previously paired with bad reputations inferred that these individuals would be less likely to cooperate (Figure 6). These results support Prediction 2 and are consistent with our interpretation that the vlPFC may be involved in negative evaluation.

**Table 1:** **Fit indices for Models 1 and 2**.

	AIC	BIC	Deviance
Model 1	−83.011	−70.859	−95.011
Model 2	−80.202	−63.999	−96.202	${\text{χ}}_{\text{(2)}}^{\text{2}}$ = 1.191, *p* = 0.5513

**Table 2:** **Fixed- and random-effects estimates for Model 1**.

Fixed-Effects Estimates
*γ*_00_ (Intercept)	0.071, (0.029, 0.113)
*γ*_01_ (SESS)	−0.143, (−0.187, −0.099)
*γ*_10_ (DISTRUST_PRE)	0.004, (−0.268, 0.277)
*γ*_20_ (ROI_ACT)	0.214, (0.014, 0.419)
**Random-Effects Estimates**
*σ_u_*	0.080, (0.040, 0.114)
*σ*	0.082, (0.063, 0.107)

*Note: The values are point estimates with 95% confidence intervals (in brackets)*.

We also performed linear model analysis to examine whether vlPFC activity *during reputation learning* in response to the faces paired with bad reputations could explain distrust toward them in the subsequent action inference tasks. Results showed that vlPFC activity during reputation learning was not significantly related to distrust in either the retrieval (*β* = 0.143, *p* = 0.356) or disregard (*β* = −0.055, *p* = 0.770) sessions. Although descriptive, vlPFC activity had less individual variability during reputation learning (coefficient of variability = 0.922) than it did during action inference (3.086 and 1.910 in the retrieval and disregard sessions, respectively), which might obscure its relationship with the behavioral measure.

## Discussion

The present study yielded two main findings. First, a region of the left vlPFC was activated when participants were informed of the bad reputations of stimulus people, and more importantly, this same region was also activated by the subsequent encounters with those people, irrespective of whether participants attempted to retrieve or disregard those bad reputations. Second, in the both retrieval and disregard sessions of action inference, participants who showed greater activity of the vlPFC in response to faces that had been paired with bad reputations were more likely to infer that those people would not cooperate. These results overall are consistent with the idea that negative evaluations, which are here assumed to be related to vlPFC activity, are transferred from a bad reputation to the target person, and consequently, the person may continue to be distrusted irrespective of the validity of their prior reputation.

The vlPFC receives multimodal sensory information about external stimuli including visual, auditory, and somatosensory inputs (Price, 2008; Romanski, 2012). In addition, this region is contiguous with and interconnected to the orbitolateral prefrontal cortex representing internal signals about visceral reactions (Öngür and Price, 2000; Price, 2008). Visceral reactions can function as a signal of the emotional meaning or value of a certain stimulus for the organism, as they convey information regarding the demands of the organism to maintain homeostasis and satisfy basic needs (Damasio et al., 1996; Craig, 2002). The vlPFC may thus be able to associate the sensory representations of an external stimulus with its value. Activation in this region upon perceiving an external stimulus may reflect the decoding of the stimulus’ associated value (i.e., evaluation of the stimulus). In fact, the vlPFC shows greater activation to emotional than neutral stimuli regardless of the stimulus type (i.e., faces and scenes; Sabatinelli et al., 2011) and has been reported to be particularly responsive to negative stimuli (Vytal and Hamann, 2010; Mende-Siedlecki et al., 2013). These findings support our interpretation that vlPFC activity in the present study is related to negative evaluation. Our arguments, which are subject to the problem of reverse inference (Poldrack, 2006), are corroborated by the data showing that vlPFC activity explained the negative evaluations of people with bad reputations.

One may argue that activity in the vlPFC during our action-inference tasks reflects participants’ attempts to recall the reputations that had been paired with stimulus faces, rather than the activation of negative evaluations associated with the stimuli. In fact, it has been proposed that the vlPFC contributes to cognitive control of memory, the volitional retrieval and selection of task-relevant knowledge (Badre and Wagner, 2007). This interpretation, however, does not easily account for some aspects of the present data. First, vlPFC activity was not greater in the retrieval than in the disregard sessions. Behavioral results indicate that participants actually attempted not to retrieve the memorized reputations in the disregard session. Therefore, if the vlPFC was involved in the recall of reputations, it should have been less active during the disregard than retrieval sessions. Second, in both sessions, vlPFC activity was positively related to inferences congruent with the memorized reputations. If vlPFC activity reflected the recall of the memorized reputations, it should have been positively and *negatively* related to the inferences congruent with the reputations in the retrieval and disregard sessions, respectively, since the recall of reputations enables conscious correction of their biasing influences on judgments in the disregard session (Sweldens et al., 2010; Balas and Gawronski, 2012). These arguments against the alternative account, however, are admittedly not decisive. The action inference task is limited in that faces are always presented in the context of a judgment. It is important to demonstrate that the mere presentation of faces can activate the vlPFC after reputation learning to gain further support for the evaluation transfer view. Thus, a promising procedure for the future experiment might be to measure vlPFC activity while participants are not engaging in any evaluative task and relate it to subsequent evaluations.

Our claim for the involvement of the vlPFC in evaluation transfer does not preclude the possible role of conscious recall of reputations in performing the action inference task. Although speculative, longer RTs during the retrieval session as compared to the other sessions suggests that the performance was at least partly based on time-consuming, effortful retrieval processes. This might explain why in the conjunction analysis, vlPFC activity in the bad-reputation condition was detected only when a lenient statistical threshold was used. That is, in our data, learned associations between faces and bad reputations might not have been strong enough to robustly activate negative evaluation upon viewing faces alone, thereby resulting in only moderate vlPFC activity during action inference. Although we demonstrated significant vlPFC activity in ROI analysis, it would be important to see whether the results could be replicated with more conservative conjunction analysis when using a more intensive reputation learning task.

In addition to conjunction analysis, we were unable to obtain significant results from the analysis on the relationship between vlPFC activity during reputation learning and behavioral performance in action inference tasks. It is possible that the relationship might be masked by low individual variability in vlPFC activity during reputation learning. The low variability might make sense considering that the vlPFC ROI was selected using the data on reputation learning (i.e., the ROI contained only those voxels showing large signals during reputation learning), and that a bad reputation on embezzlement would be evaluated negatively by everyone. Thus, the use of a variety of (bad) reputations as in previous studies (Schiller et al., 2009; Bell et al., 2012; Mende-Siedlecki et al., 2013) could introduce more variability in vlPFC activity, which might enable detection of the relationship between vlPFC activity during reputation learning and subsequent distrust.

Another major concern regarding the evaluation transfer model is that the activity of evaluation-related limbic structures other than the vlPFC was not detected in this study. In particular, we had expected greater activity of the anterior insula in response to bad reputations considering its possible involvement with anger and disgust (Murphy et al., 2003; Sanfey et al., 2003; Rilling et al., 2008). The role of the amygdala in the evaluation of others’ behaviors has also been highlighted (Buckholtz et al., 2008; Schiller et al., 2009). As mentioned in the “Results” Section, participants might have perceived the no-reputation condition as risky or ambiguous because they were unsure about whether the stimulus individuals in this condition had returned or embezzled the money. The evaluation-related regions listed above are known to be sensitive to risk and ambiguity (Adams et al., 2003; Singer et al., 2009). In fact, conjunction analysis revealed that these regions were activated not only in the good- and bad-reputation conditions but also in the no-reputation condition. Thus, the subtraction between the good-/bad- and no-reputation conditions could have cancelled out their activity. The use of a more neutral baseline condition in future research may elucidate the roles of those structures in evaluation transfer.

The role of valence in evaluation transfer also remains in need of being carefully examined. Our findings that bad reputations persisted while good ones did not might reflect a human sensitivity to cheaters that has evolved as an adaptation to secure reciprocity in social exchange (Cosmides and Tooby, 1989). However, any brain region including the vlPFC did not show greater activation for bad than for good reputations. In addition, although speculative, cheating behaviors would be perceived as not only negative but also uncommon (Mende-Siedlecki et al., 2013). It is therefore possible that the bad reputations for having embezzled the money were so unexpected that they captured the attention of participants (Bell and Buchner, 2012), facilitating evaluation transfer and the formation of persistent memory. In this study, we controlled for the effect of unexpectedness by means of *post hoc* parametric modulation, and one may doubt the validity of the specific functional assumption in the analysis. We would like to note that the model without the parametric modulation regressors reproduced the vlPFC responses to the faces paired with bad reputations and their relation to distrust, and thus, these main findings should not be affected by the arbitrariness of the presented model. Nevertheless, future research should experimentally manipulate the unexpectedness of good and bad reputations in order to clarify the effect of valence on evaluation transfer. In addition, reputation learning is known to vary across the lifespan (Fett et al., 2014), and therefore, our results from a young-adult population might not generalize to other ages. It would be especially important to determine the changes in later life given the possible vulnerability of older adults to cheating (Castle et al., 2012).

Finally, the formation of stimulus-value associations has been extensively studied using computational models of reinforcement learning (O’Doherty, 2004; Behrens et al., 2009). As our experimental tasks did not clearly involve reinforcement (i.e., participants did not receive either reward or punishment), this paper is written within a descriptive framework of evaluative conditioning, the role of which in attitude formation has been highlighted in the social psychology literature (Fazio, 2007; Jones et al., 2010). Nevertheless, considering that reinforcement values could be defined for any type of stimuli (e.g., pictures; Katahira et al., 2011), it would be interesting to apply computational modeling to reputation learning. To achieve this goal, a reputation learning task might need to include fewer stimulus persons and more learning opportunities (e.g., Chang et al., 2010) so that reliable estimation is possible.

In conclusion, this study demonstrated that once participants had learned a stimulus face’s bad reputation, they came to activate the vlPFC when judging that face, independent of the validity of the bad reputation. In addition, vlPFC activity explained the participants’ distrust toward such people. Taken together with the implicated role of the vlPFC in negative evaluation, the findings are interpreted as reflecting evaluation transfer that directly associates the negative evaluation of a bad reputation with a target person. Our results advance a possible neurocognitive explanation as to why bad reputations continue to affect judgments even after they have been shown to be invalid.

## Author Contributions

AS designed and performed experiments, analyzed data and wrote the article. YI, SK, and MK performed experiments. HO, JK, and HCT gave technical support and conceptual advice. TN performed experiments and gave technical support and conceptual advice. All authors discussed the results and implications and commented on the manuscript at all stages.

## Conflict of Interest Statement

The authors declare that the research was conducted in the absence of any commercial or financial relationships that could be construed as a potential conflict of interest.

## Footnotes

^1^This does not mean that the US becomes detached and free from the evaluation. Thus, although the term “transfer” is commonly used, “generalization” or “contagion” of evaluation might be less confusing wording. Jones et al. (2009) have argued that the transfer arises without awareness and so coined the term “implicit misattribution.” The current study did not disentangle conscious and unconscious learning, and so we use the more general term “evaluation transfer.”

^2^Perfect performance in the baseline session of action inference precluded parametric modulation analysis of the fMRI data described later (see “fMRI Data Analysis” Section).

^3^The remaining 28 participants did not suffer from mental health problems (self-report). Six of them majored in psychology, and may have had some insight into the experimental procedure involving deception. However, exclusion of their data did not change the main results.

^4^The rest was included to minimize fatigue during the long experiment. In order to assist the alignment of head positions before and after the rest, three colored marks were placed on the face of the participant (between the eyes, the left temple, and the middle of the forehead), and the locations of these marks relative to the head coil were kept as consistent as possible.

^5^It would be reasonable to assume that the unexpectedness decreases as face-reputation pairs are repeated. In this study, however, the unexpectedness did not seem to reach zero even in the last (third) run, given that participants’ performance on the retrieval session of the action inference task was not perfect (accuracy: *M* ± *SD* = 0.771 ± 0.125). We thus chose the specific functional form described above to model the decrease of unexpectedness (i.e., it was assumed to decrease by 75% from the first to the last run). This assumption may appear arbitrary, but the following results were essentially unchanged even when analyzing the data assuming that the unexpectedness was constant across runs or when removing the parametric modulation regressors. Thus, our findings were not affected by the specific assumption.

^6^Thus, ROI selection and analysis were performed using different datasets of different tasks, mitigating problems caused by non-independent data (Kriegeskorte et al., 2009). It is still possible, however, the two datasets have some dependency due to, e.g., common between-participant variance (Poldrack and Mumford, 2009).

^7^Session-specific intercepts were assumed for both models because DISTRUST_POST was expectedly smaller in the disregard than retrieval sessions (Suzuki et al., 2013).

^8^More specifically, linear mixed model analysis was performed using the following model: vlPFC activity_si_ = *β*_0_ + *β*_1_ × Session_s_ + *u_i_* + *ε_si_*, with *s* denoting the session of action inference (1 = retrieval session, 2 = disregard session), *i* denoting the participant (*i* = 1, …, 28), Session being a dummy variable (Session_1_ = −1, Session_2_ = 1), *β*_0_ representing the average of the vlPFC activity across sessions, *β*_1_ representing the difference in the vlPFC activity between the two sessions, and *u_i_* and *ε_si_*, respectively, indicating random effects associated with each participant and each observation. The estimates of the fixed effects were *β*_0_ = 0.562 (*p* = 0.006, 95% CI [0.186, 0.937]) and *β*_1_ = 0.163 (*p* = 0.338, 95% CI [−0.170, 0.496]).

^9^The other regions that were identified by the conjunction analysis were the left superior prefrontal cortex (20 voxels, [−10, 44, 50], *Z* = 2.740), the right superior temporal sulcus (54 voxels, [56, −24, −4], *Z* = 2.219), and the left medial prefrontal cortex (31 voxels, [−4, 52, 16], *Z* = 2.093). In this article, the ROI specification was performed with the data during reputation learning (i.e., Figure 4) rather than with this conjunction analysis in order to avoid the criticism of “double dipping” (Kriegeskorte et al., 2009; Vul et al., 2009) in the following ROI analysis (see also footnote 6).
